# The gastrointestinal tract is a major source of the acute metformin-stimulated rise in GDF15

**DOI:** 10.1038/s41598-024-51866-2

**Published:** 2024-01-22

**Authors:** John W. R. Kincaid, Debra Rimmington, John A. Tadross, Irene Cimino, Ilona Zvetkova, Arthur Kaser, Paul Richards, Satish Patel, Stephen O’Rahilly, Anthony P. Coll

**Affiliations:** 1 Institute of Metabolic Science, University of Cambridge, Cambridge, CB2 0QQ UK; 2 Harvard Medical School, Boston, MA 02115 USA; 3 Cambridge Genomics Laboratory, Cambridge University Hospitals NHS Foundation Trust, Cambridge, CB2 0QQ UK; 4 NHS East Genomic Laboratory Hub, East Genomics, Cambridge, CB2 0QQ UK; 5 Department of Histopathology, Cambridge University Hospitals NHS Foundation Trust, Cambridge, CB2 0QQ UK; 6 Cambridge Institute of Therapeutic Immunology and Infectious Disease, Jeffrey Cheah Biomedical Centre, University of Cambridge, Cambridge, CB2 0AW UK; 7 Division of Gastroenterology and Hepatology, Department of Medicine, University of Cambridge, Addenbrooke’s Hospital, Cambridge, CB2 0QQ UK; 8 Kallyope, Inc., 430 East 29th, Street, New York, NY 10016 USA

**Keywords:** Obesity, Feeding behaviour, Hypothalamus, Endocrine system and metabolic diseases

## Abstract

The hormone GDF15 is secreted in response to cellular stressors. Metformin elevates circulating levels of GDF15, an action important for the drug’s beneficial effects on body weight. Metformin can also inhibit mammalian respiratory complex I, leading to decreases in ATP:AMP ratio, activation of AMP Kinase (AMPK), and increased GDF15 production. We undertook studies using a range of mice with tissue-specific loss of *Gdf15* (namely gut, liver and global deletion) to determine the relative contributions of two classical metformin target tissues, the gut and liver, to the elevation of GDF15 seen with metformin. In addition, we performed comparative studies with another pharmacological agent, the AMP kinase pan-activator, MK-8722. Deletion of *Gdf15* from the intestinal epithelium significantly reduced the circulating GDF15 response to oral metformin, whereas deletion of *Gdf15* from the liver had no effect. In contrast, deletion of *Gdf15* from the liver, but not the gut, markedly reduced circulating GDF15 responses to MK-8722. Further, our data show that, while GDF15 restricts high-fat diet-induced weight gain, the intestinal production of GDF15 is not necessary for this effect. These findings add to the body of evidence implicating the intestinal epithelium in key aspects of the pharmacology of metformin action.

## Introduction

Metformin is the world’s most widely prescribed pharmacotherapy for Type 2 diabetes mellitus (T2DM). In addition to improving glycemic control, metformin causes modest, clinically-relevant weight loss^1^.

Metformin’s effects on energy balance have been linked to growth differentiation factor 15 (GDF15)^2,3^. A member of the TGFβ superfamily, GDF15 is a stress-responsive peptide hormone produced in peripheral tissues that acts centrally via its hindbrain-localized receptor, GFRAL-RET, to cause a reduction in food intake and body weight^4,5^. We and others have identified GDF15 as a mediator of metformin-induced weight loss in mice^3,6,7^.

Metformin’s actions continue to be the subject of intense scrutiny, with the drug reported to affect multiple biochemical, cellular, and endocrine processes across a variety of tissues^8,9^. For example, evidence has shown metformin to be an inhibitor of mammalian respiratory complex I^8–10^. Through its effect on ATP synthesis/oxidative phosphorylation, metformin causes activation of AMP kinase (AMPK), a cellular energy sensor known to increase glucose uptake and fatty acid oxidation^10^. AMPK has therefore been proposed as one of the mediators of the drug’s glucoregulatory effects^11^.

In addition, debate remains regarding the tissues and cell types where metformin exerts its actions. Most of the studies attempting to identify a specific biochemical effect of metformin, whether on AMP kinase^12^, mitochondrial glycerophosphate dehydrogenase^13^, or other biochemical processes^11^, have focused on the liver. Other evidence has suggested that, rather than the liver, the intestine might be the most important site of metformin action^2^. We and others have shown that metformin increases *Gdf15* expression in the gastrointestinal (GI) tract and kidney, but not liver, of metformin-treated mice^3,4^. However, the relative contributions of different tissues to the metformin-stimulated rise in serum GDF15 levels remain uncertain.

Here, we employed a range of genetically-modified mouse models of tissue-specific *Gdf15* deletion to identify the main sources of metformin-induced increases in circulating GDF15. We also performed comparative studies utilizing a systemic pan-AMPK activator compound, MK-8722^5^, to assess whether this agent stimulates GDF15 production and secretion through targeting different tissues. Finally, as previous work has suggested that diet affects the metformin-mediated rise in circulatory GDF15^3,4,6^, we performed studies in tissue-specific *Gdf15*-knockout animals using both a high-fat diet (HFD) and a high-fat high-sucrose (HFHS) diet.

## Results

### Gut-targeted *Gdf15* ablation results in significant reductions of metformin-stimulated circulating GDF15, a response altered by antecedent diet

Gdf15^flox/flox^-Villin-Cre^+^ (*Gdf15*-gut-KO) mice were generated using Cre-Lox recombination under the control of the Villin promoter (Villin-Cre). *Cre* RNAscope in *Gdf15*-gut-KO mice showed the expected cytoplasmic RNA staining pattern in enterocytes (Supplementary Fig. A.1B). Additionally, a single spot was present in the nucleus of almost all cells, including lymphoid (Supplementary Fig. A.1B) and renal (Supplementary Fig. A.1E) cells, consistent with hybridization to the genomic *Cre* locus. To confirm and further investigate potential low level *Cre* expression in the kidney, DNase pre-treatment was used to remove genomic DNA prior to RNAscope. Nuclear hybridization was absent following DNase, while cytoplasmic mRNA signals were retained. No significant expression of *Cre* RNA was detected in the kidney following DNase treatment (Supplementary Fig. A.1C,F).

To validate loss of *Gdf15* expression in the GI tract, we analyzed the colons and kidneys from sham- and metformin-treated wild-type and *Gdf15*-gut-KO mice via quantitative PCR (Supplementary Fig. A.2). *Gdf15* expression was negligible in colon and kidney tissue from sham-treated, high-fat diet-fed wild-type and *Gdf15*-gut-KO mice (Supplementary Fig. A.2A,B,E,F). Metformin administration resulted in heightened *Gdf15* expression in the colon and kidney of WT animals, whereas in *Gdf15*-gut-KO mice a strong signal was seen in renal tissue and was absent in the gut (Supplementary Fig. A.2C,D,G,H).

To assess the effect of gut-selective *Gdf15* deletion on metformin-induced GDF15 production, as well as the impact of antecedent diet on this response, WT and *Gdf15*-gut-KO mice were fed either standard chow, a 60% HFD, or an HFHS diet for 4 weeks before receiving an acute dose of metformin.

**Figure 1:**
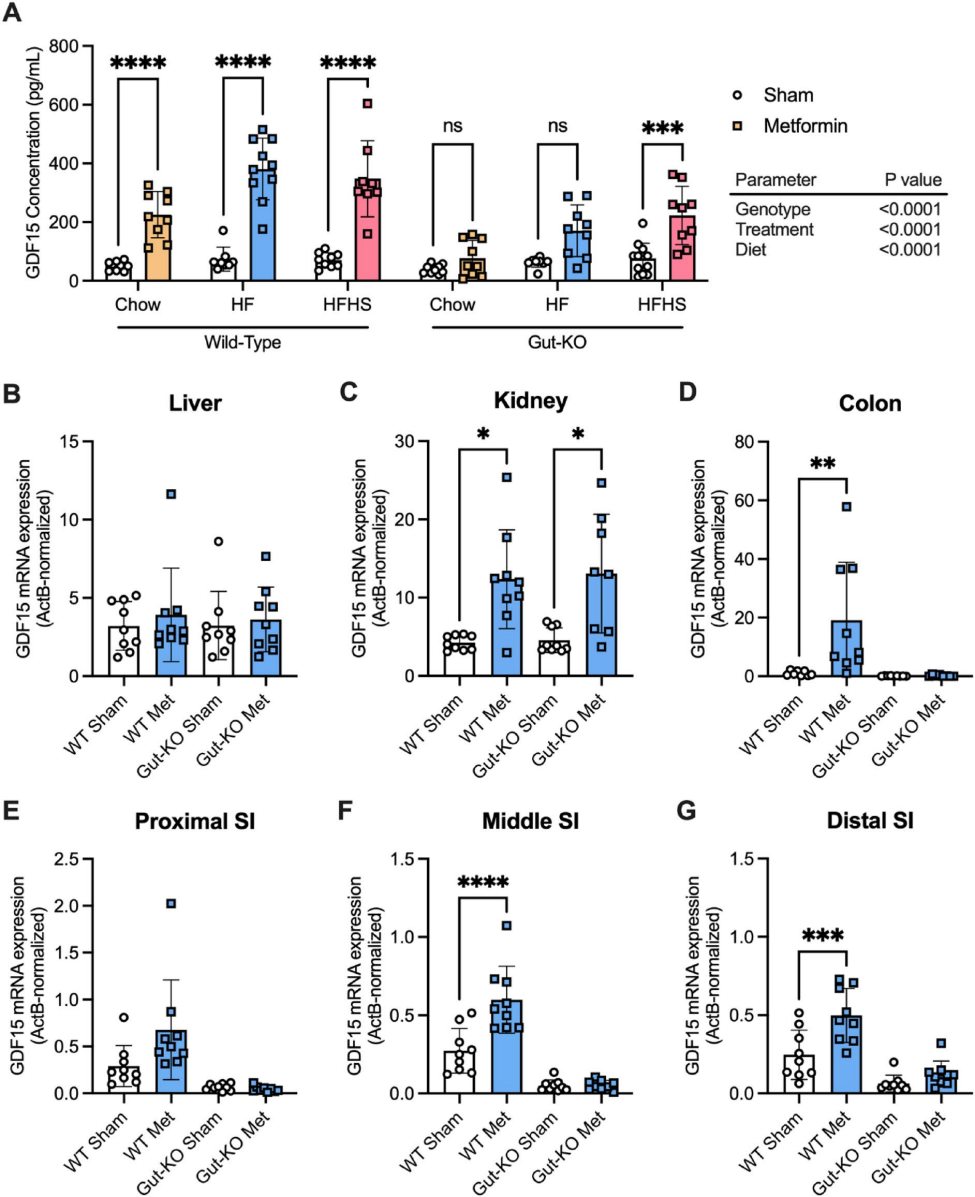
Gut-targeted *Gdf15* ablation results in significant reductions of acute metformin-stimulated circulating GDF15, a response altered by antecedent diet. (**A**) Serum GDF15 levels (mean ± SEM) in 12-week-old wild-type and *Gdf15*-gut-KO mice fed either a standard chow (Chow), 60% high-fat (HF), or high-fat high-sucrose (HFHS) diet for 4 weeks followed by a single oral dose of water (sham) or 600 mg/kg metformin, n = 9/group, and ns = non-significant, ***p < 0.001, and ****p < 0.0001 as determined by three-way ANOVA. (**B**–**G**) *Gdf15* mRNA expression (normalized to expression levels of actin B) in the (**B**) liver, (**C**) kidney, (**D**) colon, (**E**) proximal small intestine, (**F**) middle small intestine, and (**G**) distal small intestine from 60% high-fat diet-fed wild-type and *Gdf15*-gut-KO mice 6 h after receiving a single oral dose of water (Sham) or 600 mg/kg metformin (Met). n = 9/group, mean ± SEM, and *p < 0.05, **p < 0.01, ***p < 0.001, and ****p < 0.0001 as determined by two-way ANOVA.

In WT mice, as expected, metformin significantly increased circulating GDF15 levels, with the absolute levels measured being higher in mice fed high-calorie (HF and HFHS) diets (Fig. 1A). In contrast, in mice lacking *Gdf15* specifically in the gut, the circulating GDF15 response to metformin was significantly diminished. Specifically, on standard chow, the levels achieved in *Gdf15*-gut-KO animals were ~ 34% of those seen in WT animals, and on HFD, the metformin stimulated GDF15 rise in *Gdf15*-gut-KO mice was only ~ 40% of that of WT mice. Interestingly, the HFHS diet was the only dietary paradigm in which metformin treatment elicited a significant rise in circulating GDF15 in *Gdf15*-gut-KO mice, although, as with all previous dietary manipulations, there was a difference in the magnitude of the rise in GDF15 levels between WT and *Gdf15*-gut-KO animals.

qPCR was performed on isolated kidney, liver, and sectioned small intestinal and colonic tissues from all three dietary groups. In WT animals fed an HFD, in keeping with previous results^3^, significant increases in *Gdf15* mRNA were observed following metformin treatment in the kidney, colon, and small intestine. There was no such increase in the liver (Fig. 1B-G). The results of chow-fed (Supplementary Fig. A.3) and HFHS-fed (Supplementary Fig. A.4) tissue panels emulated the *Gdf15* expression pattern in HFD-fed mice, and unchanged hepatic expression of *Gdf15* post-metformin administration was displayed in both WT and *Gdf15*-gut-KO animals.

To further characterize the global and gut-specific *Gdf15* knockout models, we measured circulating levels of GDF15 and GLP-1 following a lower dose (300 mg/kg) of metformin comparable to previous studies^3,6^. Compared to wild-type mice, there was no significant rise in GDF15 in *Gdf15*-gut-KO animals (Supplementary Fig. A.5A) while there was a significant rise in GLP-1 observed across all genotypes (Supplementary Fig. A.5B). Similarly, the response in PYY to an acute higher dose (600 mg/kg) of metformin was identical across genotypes (Supplementary Fig. A.5C).

### Loss of liver *Gdf15* does not impact the acute metformin-stimulated rise in circulatory GDF15

**Figure 2:**
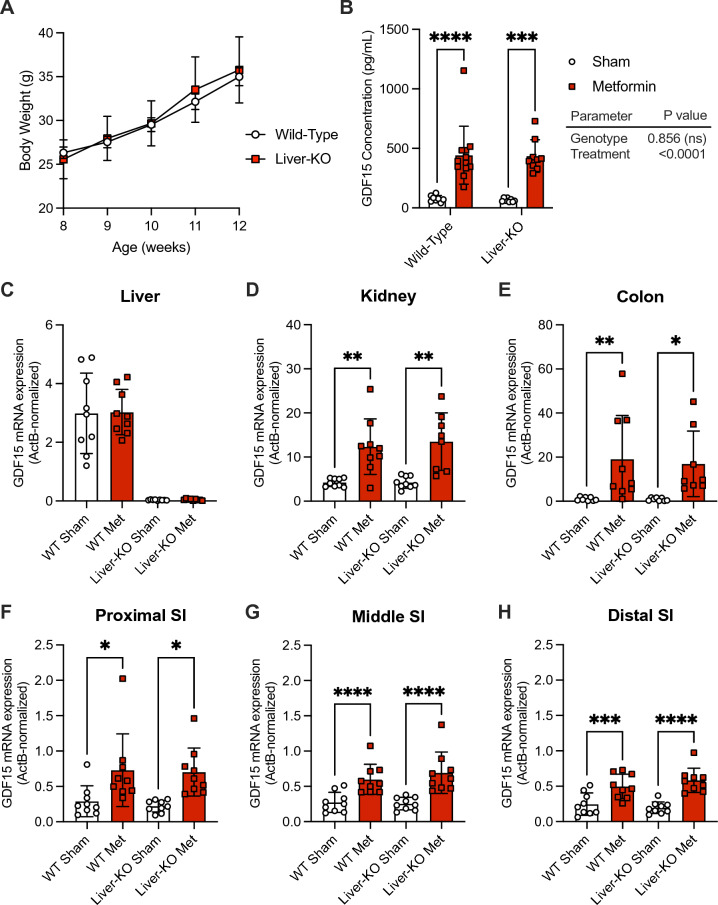
Loss of liver *Gdf15* does not impact the acute metformin-stimulated rise in circulatory GDF15. (**A**) Body weight of 12-week-old wild-type (n = 17) and *Gdf15*-liver-KO (n = 10) mice on a 60% high-fat diet for 4 weeks beginning at 8 weeks of age, mean ± SEM. (**B**) Serum GDF15 levels (mean ± SEM) in 12-week-old wild-type and *Gdf15*-liver-KO mice fed a 60% high-fat diet for 4 weeks followed by a single oral dose of water (sham) or 600 mg/kg metformin, n = 9/group, and ns = non-significant, ***p < 0.001, and ****p < 0.0001 as determined by two-way ANOVA. (**C**–**H**) *Gdf15* mRNA expression (normalized to expression levels of actin B) in the (**C**) liver, (**D**) kidney, (**E**) colon, (**F**) proximal small intestine, (**G**) middle small intestine, and (**H**) distal small intestine from 60% high-fat diet-fed wild-type and *Gdf15*-liver-KO mice 6 h after receiving a single oral dose of water (Sham) or 600 mg/kg metformin (Met). n = 9/group, mean ± SEM, and *p < 0.05, **p < 0.01, ***p < 0.001, and ****p < 0.0001 as determined by two-way ANOVA.

We used a *Gdf15*-liver-KO mouse model (*Gdf15*^flox/flox^-Albumin-Cre^+^) to explore the liver’s contribution to the GDF15 rise post-metformin. *Gdf15*-liver-KO mice displayed similar trends in body weight gain relative to WT mice when fed an HFD for 4 weeks (Fig. 2A). Loss of hepatic *Gdf15* had no impact on response to acute metformin, with an identical rise in GDF15 seen in WT and *Gdf15*-liver-KO mice (Fig. 2B). Further, *Gdf15* expression profiles in the kidney and the gut were identical in both genotypes (Fig. 2C-H).

### AMPK activator treatment and GDF15

**Figure 3:**
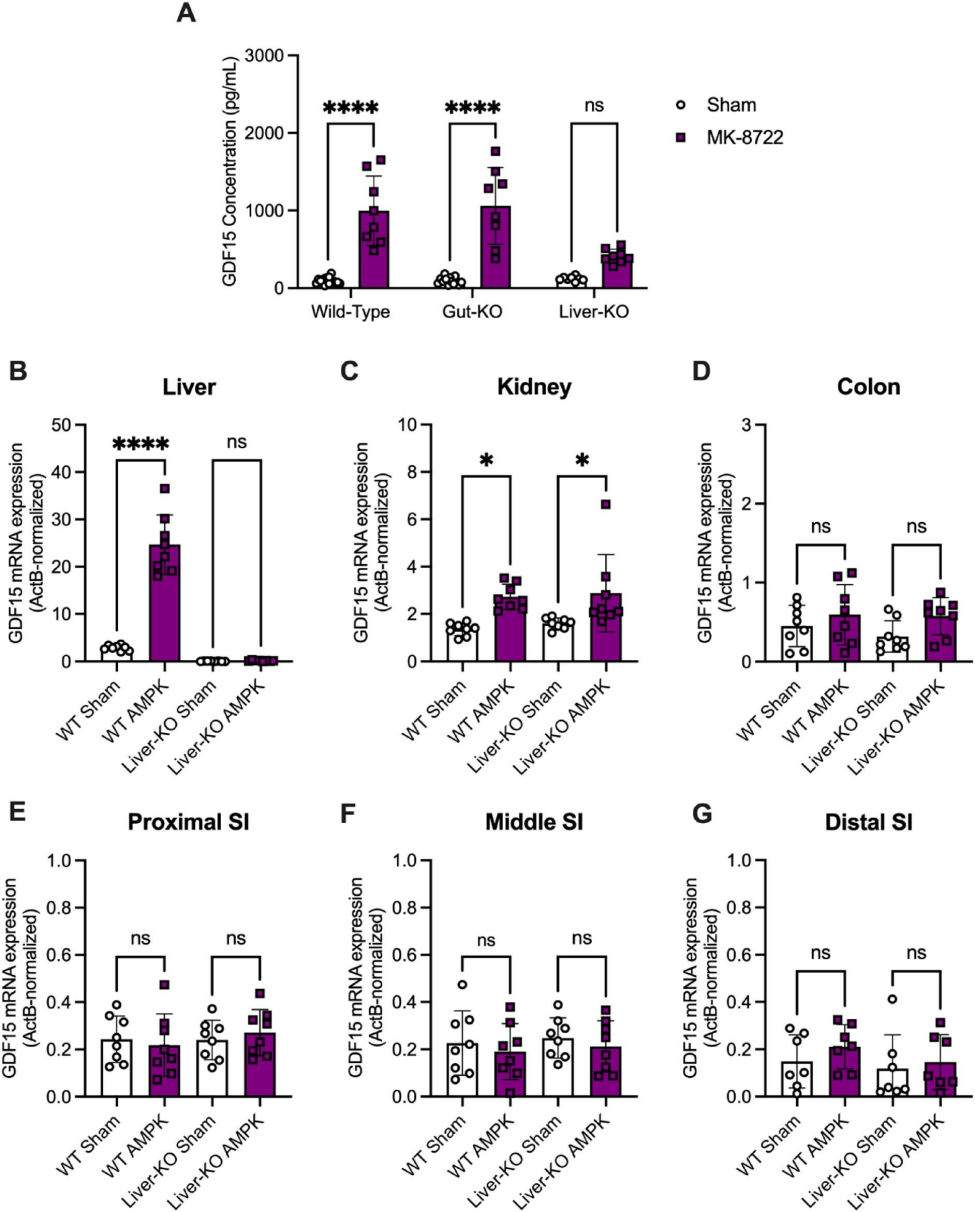
The liver is a major contributor to the increase in circulating GDF15 in response to oral administration of an AMPK activator. (**A**) Serum GDF15 levels (mean ± SEM) in 20-week-old wild-type and *Gdf15*-liver-KO mice fed a 60% high-fat diet for 4 weeks followed by a single oral dose of water (sham) or 30 mg/kg MK-8722, n = 8/group, mean ± SEM, and ns = non-significant and ****p < 0.0001 as determined by two-way ANOVA. (**B**–**G**) *Gdf15* mRNA expression (normalized to expression levels of actin B) in the (**B**) liver, (**C**) kidney, (**D**) colon, (**E**) proximal small intestine, (**F**) middle small intestine, and (**G**) distal small intestine from high-fat diet-fed wild-type and *Gdf15*-liver-KO mice 4 h after receiving a single oral dose of water (Sham) or 30 mg/kg MK-8722. n = 8/group, mean ± SEM, and ns = non-significant, *p < 0.05, and ****p < 0.0001 as determined by two-way ANOVA.

Having identified the gut as a major source of metformin-stimulated GDF15 production, we sought to assess whether other pharmacologic agents of GDF15 induction act through different organs. AMPK activation has been shown to elevate circulatory GDF15 levels through an increase in hepatic *Gdf15* expression^7^, so we administered an AMPK activator, MK-8722, to our tissue-specific *Gdf15*-knockout mouse models (Fig. 3).

Although MK-8722 treatment increased circulating GDF15 ~ 10.7-fold in WT and *Gdf15*-gut-KO mice, *Gdf15*-liver-KO animals displayed a diminished (~ 3.4-fold) rise in GDF15 (Fig. 3A). In WT mice, significant increases in *Gdf15* mRNA were observed following MK-8722 treatment in the liver (Fig. 3B) and kidney (Fig. 3C), while *Gdf15* expression throughout the small intestine and colon remained unaffected by MK-8722 administration (Fig. 3D–G) independent of genotype. Together, these data identify the liver as the major contributor to the rise in serum GDF15 levels elicited by the AMPK activator, MK-8722, with the kidney also contributing to this response.

### The gut does not contribute significantly to the protective effect of GDF15 on weight gain in response to a high fat diet

**Figure 4:**
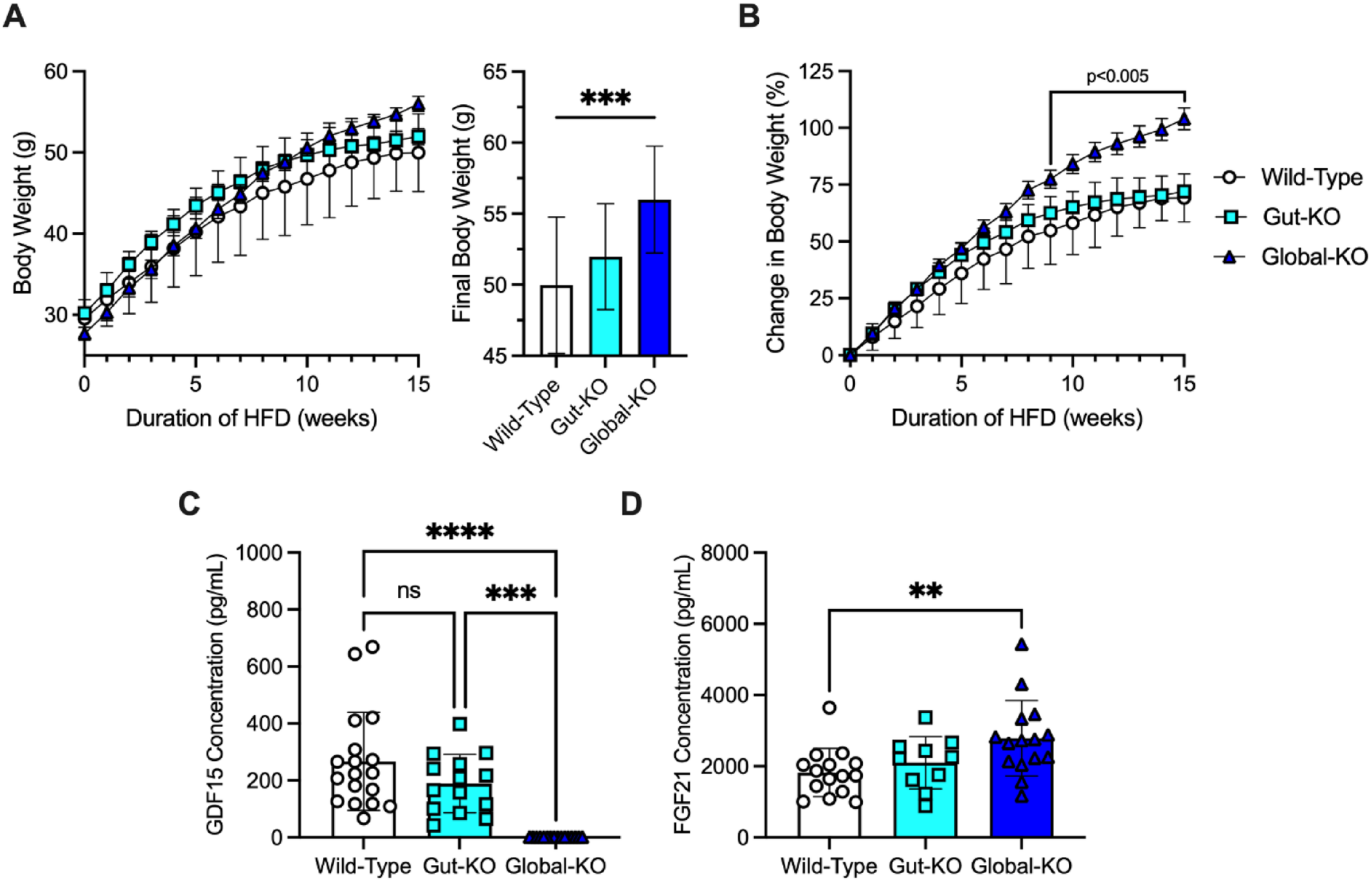
The gut does not contribute significantly to the protective effect of GDF15 on weight gain in response to a high fat diet. (**A**) Body weight of 23-week-old wild-type (n = 18), *Gdf15*-gut-KO (n = 15), and *Gdf15*-global-KO (n = 16) mice fed a 60% high-fat diet for 15 weeks beginning at 8 weeks of age, mean ± SEM. Inset, final body weight. (**B**) Percent body weight change of 23-week-old wild-type (n = 18), *Gdf15*-gut-KO (n = 15), and *Gdf15*-global-KO (n = 16) mice fed a 60% high-fat diet for 15 weeks beginning at 8 weeks of age, mean ± SEM. From 9 to 15 weeks on diet, global-KO body weight relative to wild-type mice p-value listed as determined by two-way ANOVA with multiple comparison adjustment by Tukey’s test. (**C**) Concentration of serum GDF15 (pg/mL) in 23-week-old wild-type (n = 18), *Gdf15*-gut-KO (n = 15), and *Gdf15*-global-KO (n = 16) mice fed a 60% high-fat diet for 15 weeks beginning at 8 weeks of age, mean ± SEM. ns = nonsignificant, ***p < 0.001, and ****p < 0.0001 as determined by two-way ANOVA with multiple comparison adjustment by Tukey’s test. (**D**) Concentration of serum FGF21 (pg/mL) in wild-type (n = 18), *Gdf15*-gut-KO (n = 15), and *Gdf15*-global-KO (n = 16) mice fed a 60% high-fat diet for 15 weeks beginning at 8 weeks of age, mean ± SEM. *p < 0.05 as determined by two-way ANOVA with multiple comparison adjustment by Tukey’s test.

We next characterized the effects of gut-specific *Gdf15* ablation on diet-induced body weight gain in WT, *Gdf15*-gut-KO, and *Gdf15*-global-KO animals fed a 60% HFD for 15 weeks (Fig. 4). Whilst global deletion of *Gdf15* resulted in increased body weight gain, gut-specific GDF15 deficiency did not affect body weight trends on a long-term high-fat diet (BW at 15 weeks on a HFD WT vs *Gdf15*-gut-KO vs* Gdf15*-global-KO; 49.9 ± 4.8 g vs 51.9 ± 3.7 g vs 56.0 ± 3.8 g, respectively, *p* = 0.0003) (Fig. 4A,B).

Finally, chronic overfeeding is known to increase both circulating GDF15 and FGF21 in mice^14^. Wild-type mice fed a 60% fat diet had elevated levels of serum GDF15 compared to chow-fed mice (which exhibit basal levels of ~ 50–80 pg/mL (Fig. 1A)). Gut-selective *Gdf15* ablation resulted in a slight, albeit nonsignificant decrease in circulating GDF15 levels relative to WT mice (Fig. 4C). Consistent with the findings of Patel et al.^15^, HFD-fed *Gdf15*-global-KO mice had significantly higher FGF21 levels than HFD-fed WT littermates (Fig. 4D).

## Discussion

The reduction of body weight that occurs in response to metformin, though modest, contributes significantly to its beneficial impact on metabolic health^1^. Several^3,6,16^ but not all^4^ independent studies have reported that the rise in circulating GDF15 in response to metformin is necessary for its effects on energy balance and body weight. These studies have variously highlighted the gut^3^, the liver^6^, and the kidney^3,16^ as important sources of GDF15 production in response to metformin. Herein, we have studied mice in which *Gdf15* expression has been tissue-specifically disrupted and conclude that, when metformin is acutely administered by oral gavage the gut is the main source of the GDF15 response, with the kidney, but not the liver, also making a significant contribution.

Once it has reached the gut, metformin enters enterocytes through organic cation transporters PMAT and OCT1. Bailey et al. reported that metformin concentrations in the jejunum are ~ 30- to ~ 300-fold higher than plasma concentrations^17^. After its absorption, metformin goes to the liver via the hepatic portal circulation. Hepatic metformin concentrations have been shown to be ~ two- to ~ fourfold greater than that of the plasma in rats^18^ and mice^19^, a finding reflected by similar measurements in humans^20^. Metformin is not metabolized and is instead rapidly taken up by the kidneys and excreted unchanged in the urine. The concentration of metformin reached in renal tubules has been approximated to be ~ eightfold greater than plasma concentrations^20^.

A growing body of evidence has implicated disruption of mitochondrial function as an important mechanism underlying the beneficial metabolic effects of metformin^8–10^. Bridges et al. recently reported the structural basis for metformin’s inhibition of mammalian respiratory Complex I^10^. Given that there is much evidence supporting mitochondrial dysfunction as a strong stimulus for increases in *GDF15* expression^21–23^, this action of metformin seems to be a plausible mechanism through which it increases GDF15^24^.

Metformin has also been shown to activate AMP kinase by increasing AMP:ATP and ADP:ATP ratios^12,25–27^, although whether or not this effect is necessary for metformin to elicit its metabolic benefits remains under contention^28–30^. We found that a single, oral dose of an active AMP kinase activator elevated *Gdf15* expression in the liver and kidney, but not the gut. The apparent absence of effect on gut-derived GDF15 levels, despite the fact that it must pass through those cells, may be seen as evidence that AMPK activation in enterocytes does not increase GDF15 levels. However, we have not looked specifically at intestinal AMPK activation after MK-8772 and are aware of previous reports from different model systems that intestinal AMPK activation can have a wider impact upon metabolism, for example, through altering hepatic glucose production^31^. We have not pursued longer-term studies of either metformin or MK-8772 and it may be the case that, with chronic administration, different patterns of tissue contribution would arise. Further, the majority of these studies used high doses of metformin and included only male mice, decisions based on previous studies as a way to robustly challenge the system under investigation. Again, other dosing regimens over longer time courses in both sexes may reveal different patterns of expression.

We also further characterized the impact of dietary status on the metformin-induced GDF15 response. High-fat feeding potentiates GDF15 responses to metformin, an effect further enhanced when high fat is combined with high sugar. While the underlying mechanism for these effects remains unclear, alterations in the gut microbiome following dietary manipulations^32,33^ and metformin treatment^34–38^ are well-defined, and may be a potential area for future exploration.

Animals lacking GDF15 have previously been reported to be more susceptible to weight gain from high-fat diets than WT mice^15,39,40^. Patel et al. recently compared mice lacking *Gdf15* from liver vs. macrophages, and concluded that hepatocytes are the major source of high-fat diet-induced GDF15^15^. We report that mice lacking *Gdf15* in the intestine are not more susceptible to gaining weight on a high-fat diet, suggesting that, under normal circumstances (i.e., in the absence of metformin) gut-derived GDF15 does not contribute significantly to the weight-restraining effects of GDF15 in the presence of high-fat feeding.

While the effects of metformin on GDF15 are important for the weight-lowering effects of the drug—and those effects contribute significantly to the ability of metformin to prevent the onset of T2DM in at-risk individuals—metformin has effects on lowering glucose and insulin that are independent of GDF15-GFRAL signaling^3^. Studies to elucidate the mechanism of metformin to reduce glucose and insulin levels have largely focused on the liver^11,13^. However, prior to the demonstration of the striking impact of metformin on enterocyte expression of *Gdf15*^3^, there has been a long history of studies suggesting the gut as a key site of metformin action^41,42^.

Using insulin-resistant obese *fa/fa* rats, Pénicaud et al. identified the digestive tract as the tissue responsible for the majority of metformin-stimulated glucose utilization^2^. Patients taking metformin have robustly increased ^18^F-FDG uptake (a marker of glucose uptake and utilization), as observed by PET-CT, in the colon and small intestine^43,44^. A formulation which delivers metformin to the lower gastro-intestinal tract with much lower systemic exposure than the normal formulation is at least as efficacious as the latter in lowering glucose and HbA1c levels^45,46^.

Further evidence supporting the concept that metformin action in diabetes is not confined to the liver is provided by Gormsen et al. in which metformin is reported to actually increase endogenous glucose production in individuals with well-controlled T2DM and in normoglycemic controls^47^. Data from a series of detailed whole body glucose metabolism studies indicate that the main effect of metformin on fasting blood glucose is more likely to be through an extra-hepatic action not in skeletal muscle, and suggested to be in the gut^47^, with the increase in EGP the result of the lowering of plasma insulin and the rise in lactate production from anaerobic glycolysis in the “first responder” tissue. Recently Tobar et al. confirmed the effects of metformin on basolateral glucose uptake in the intestine and that this triggers an increase in hepatic glucose output in normoglycemic animals. In contrast, in hyperglycemic animals they observed a reduction of hepatic glucose output (at least as assessed by pyruvate tolerance testing) that they attribute to gut-liver crosstalk, the specific nature of which is yet to be precisely defined^48^.

Given the growing realization that the enterocyte may be a major site of action of metformin, further detailed exploration of its effects on *Gdf15* expression in the gut may provide a window of opportunity for increasing understanding of the broader mechanism of action of this widely-used drug.

## Methods

### Animal studies

#### Animals

The research reported in this publication is solely the responsibility of the authors and does not necessarily represent the official views of the Medical Research Council. All mouse studies were performed at the University of Cambridge in accordance with UK Home Office Legislation regulated under the Animals (Scientific Procedures) Act 1986 Amendment, Regulations 2012, following ethical review by the University of Cambridge Animal Welfare and Ethical Review Body (AWERB). The study is reported in accordance with ARRIVE guidelines. All methods were performed in accordance with the relevant guidelines and regulations. Mice were maintained in a 12 h:12 h light:dark cycle (lights on 07:00–19:00), temperature-controlled (22 °C) facility, with ad libitum access to food (RM3(E) Expanded Chow (Special Diets Services)) and water. Any mice bought from an outside supplier were acclimatized in a holding room for at least one week before study.

Mice carrying the GDF15 knockout-first “tm1a” allele [C57BL/6N-Gdf15^tm1a(KOMP)Wtsi/H^] were obtained through the IMPC, from the MRC Harwell Production Centre. A “conditional-ready *Gdf15* tm1c” allele [C57BL/6N-Gdf15^tm1c(KOMP)Wtsi/H^] expressing mouse was generated in-house. Briefly, one-cell stage embryos (obtained from super-ovulated wild-type C57Bl/6N females fertilized in vitro with sperm from homozygous *Gdf15*^Tm1a^ male mice) were injected into the pronucleus with 100 ng/μL StemMACS Flp Recombinase mRNA (Miltenyi Biotec) then transferred into the uteri of pseudo pregnant recipient females (F1 hybrids from C57Bl/6 J female × CBA/Ca male crosses). Mice from the transfer (F1 mice) were analyzed for the presence of the *Gdf15* Tm1c and Tm1a alleles. F1 founders were then crossed twice with wild-type C657Bl/6N mice before establishing the *Gdf15* Tm1c and *Gdf15* Tm1a colonies.

The *Gdf15* Tm1c mouse model contains loxP sites flanking exon 2 of *Gdf15* gene. *Gdf15*-knockout mice can be derived from crossing mice carrying the *Gdf15* Tm1c [C57BL/6N-Gdf15^tm1c(KOMP)Wtsi/H^] allele with either mice expressing *Cre* in the germline to create global knockout or with tissue-specific *Cre*-expressing mice to create mice lacking *Gdf15* in specific tissues.

*Gdf15*-liver-deficient C57Bl/6N-Gdf15^flox/flox^-Albumin-Cre^+^ (referred to as *Gdf15*-liver-KO mice) were obtained from crossing mice carrying the *Gdf15* Tm1c allele with transgenic mice expressing *Cre* under the control of the Albumin promoter. To establish *Gdf15*-liver-KO colonies, male *Gdf15*-liver-KO mice were obtained from Satish Patel and David Savage at the University of Cambridge Wellcome-MRC Institute of Metabolic Science-Metabolic Research Laboratories. *Gdf15*-gut-deficient C57Bl/6N-Gdf15^flox/flox^-Villin-Cre^+^ (referred to as *Gdf15*-gut-KO mice) were obtained from crossing mice carrying the *Gdf15* Tm1c [C57BL/6N-Gdf15^tm1c(KOMP)Wtsi/H^] allele with Tg(Vil1-Cre)997Gum mice. Vil1-Cre mice express Cre recombinase in villus and crypt epithelial cells of the small and large intestines as previously described^49^. The Tg(Vil1-Cre)997Gum mice were obtained from The Jackson Laboratory (https://www.jax.org/strain/004586). C57Bl/6J and C57Bl/6N mice were obtained from Charles River. Sample sizes were determined based on homogeneity and consistency of characteristics in the selected models and were sufficient to detect statistically significant differences in body weight and serum parameters between groups. Experiments were performed with animals of a single sex in each study. Transgenic animals were randomised into the treatment groups based on body weight such that the mean body weights of each group were as close to each other as possible, but without using an excess number of animals. No samples or animals were excluded from analyses. Researchers were not blinded to group allocations.

#### Mouse study 1 (MS1)

##### Acute metformin and 4-week 60% high-fat diet, wild-type, *Gdf15*-liver-knockout, and *Gdf15*-gut-knockout mice

Experimental cohorts of male *Gdf15*-gut-KO and *Gdf15*-liver-KO mice were generated by GDF15^flox/flox^-Villin-Cre^-^ x GDF15^flox/flox^-Villin-Cre^+^ and C57Bl/6N-Gdf15^flox/flox^-Albumin-Cre^+^ x C57Bl/6N-Gdf15^flox/flox^-Albumin-Cre^-^ breeding pairs, respectively. Mice were approximately 8 weeks of age when transferred from a standard chow diet to a 60% high-fat diet (HFD; Research Diet Inc. no. 12492i) for 4 weeks before receiving a single dose by oral gavage of either 600 mg/kg metformin or a matched volume of vehicle (water). While fed an HFD, the body weight of each mouse was monitored weekly. GDF15^flox/flox^-Villin-Cre^-^ (wild-type), *Gdf15*-gut-KO, and *Gdf15*-liver-KO animals were randomized into the treatment groups based on body weight such that the mean body weights of each group were as close to each other as possible. Oral gavaging was performed at 9:00 AM, GMT, after which mice were returned to ad libitum 60% HFD before being culled 6 h after treatment by cervical dislocation. Blood was collected into a Starstedt Serum Gel 1.1 mL Micro Tube, left for 30 min at room temperature, then spun for 10 min at 10,000 g at 4 °C before being frozen and stored at − 80 °C until assayed. Mouse GDF15 levels were measured using a Mouse GDF15 DuoSet ELISA (R&D Systems) which had been modified to run as an electrochemiluminescence assay on the Meso Scale Discovery assay platform. Tissues were fresh frozen on dry ice and kept at − 80 °C until the day of RNA extraction.

RNA extraction was carried out with approximately 100 mg of tissue in 1 ml Qiazol Lysis Reagent (Qiagen 79306 l) using Lysing Matrix D homogenisation tube and Fastprep 24 Homogenizer (MP Biomedicals) and Qiagen RNeasy Mini Kit (no. 74106) following manufacturers’ protocols. Five-hundred nanograms of RNA was used to generate cDNA using Promega M-MLV reverse transcriptase followed by TaqMan qPCR in triplicate for *Gdf15*. Samples were normalized to *Actb*. TaqMan Probes: Mm00442228 m1 GDF15, Mm02619580_g1 ActB, TaqMan; 2 × universal PCR Master mix (Applied Biosystems Thermo Fisher, 4318157); QuantStudio 7 Flex Real time PCR system (Applied Biosystems Life Technologies).

#### Mouse study 2 (MS2)

##### Acute metformin and standard chow diet, *Gdf15*-gut-knockout and wild-type mice

Experimental cohorts of male *Gdf15*-gut-KO were generated as in MS1. Mice were approximately 12 weeks of age before receiving a single dose by oral gavage of either 600 mg/kg metformin or a matched volume of vehicle (water). GDF15^flox/flox^-Villin-Cre^-^ (wild-type) and *Gdf15*-gut-KO animals were randomized into treatment groups based on body weight such that the mean body weights of each group were as close to each other as possible. Oral gavaging was performed at 9:00 AM, GMT, and mice were culled 6 h after treatment by cervical dislocation. Blood was collected, processed, and analyzed for Mouse GDF15 as in MS1. Tissues were fresh frozen on dry ice and kept at − 80 °C until the day of RNA extraction.

#### Mouse study 3 (MS3)

##### Acute metformin and 4-week HFHS diet, *Gdf15*-gut-knockout and wild-type mice

Experimental cohorts of male *Gdf15*-gut-KO mice were generated as in MS1. Mice were approximately 8 weeks of age when transferred from a standard chow diet to a high-fat high-sucrose diet (HFHS; Research Diet Inc. no. 20022503) for 4 weeks before receiving a single dose by oral gavage of either 600 mg/kg metformin or a matched volume of vehicle (water). While fed an HFHS diet, the body weight of each mouse was monitored weekly. GDF15^flox/flox^-Villin-Cre^-^ (wild-type) and *Gdf15*-gut-KO animals were randomised into treatment groups based on body weight such that the mean body weights of each group were as close to each other as possible. Oral gavaging was performed at 9:00 AM, GMT, and mice were returned to ad libitum HFHS diet after treatment administration. 6 h after treatment administration, animals were culled by cervical dislocation. Blood was collected, processed, and analyzed for Mouse GDF15 as in MS1. Tissues were fresh frozen on dry ice and kept at − 80 °C until the day of RNA extraction. RNA extraction was carried out and quantitative PCR was performed as in MS1.

#### Mouse study 4 (MS4)

##### AMPK activation and 4-week 60% high-fat diet, wild-type, *Gdf15*-liver-knockout, and *Gdf15*-gut-knockout mice

Experimental cohorts of male *Gdf15*-gut-KO and *Gdf15*-liver-KO mice were generated as in MS1. Mice were approximately 16 weeks of age when transferred from a standard chow diet to a 60% high-fat diet (HFD; Research Diet Inc. no. 12492i) for 4 weeks before receiving a single dose by oral gavage of either a pan-AMPK activator, MK-8722 (30 mg/kg), or a matched volume of vehicle (water). MK-8722 was purchased from MedChemExpress (MCE Cat. No. HY-111363). While fed on HFD, the body weight of each mouse was monitored weekly. GDF15^flox/flox^-Villin-Cre^-^ (wild-type), *Gdf15*-gut-KO, and *Gdf15*-liver-KO animals were randomized into the treatment groups based on body weight such that the mean body weights of each group were as close to each other as possible. Oral gavaging was performed daily at 9:00 AM, GMT, after which mice were returned to ad libitum 60% HFD before being culled 4 h after treatment by cervical dislocation. Blood was collected, processed, and analyzed for Mouse GDF15 as in MS1. Tissues were fresh frozen on dry ice and kept at − 80 °C until the day of RNA extraction. RNA extraction was carried out and quantitative PCR was performed as in MS1.

#### Mouse study 5 (MS5)

##### Acute metformin and 4-week high-fat diet, wild-type, *Gdf15*-gut-knockout, and *Gdf15*-global-knockout mice

Experimental cohorts of male *Gdf15*-gut-KO and *Gdf15*-global-KO mice were generated as in MS2. Mice were between 13 and 16 weeks of age when transferred from a standard chow diet to a 60% high-fat diet (HFD; ResearchDiets no. 12492i) for 4 weeks before receiving a single dose by oral gavage of either 600 mg/kg metformin or a matched volume of vehicle (water). While fed an HFD, the body weight of each mouse was monitored weekly. GDF15^flox/flox^-Villin-Cre^-^ (wild-type), *Gdf15*-gut-KO, and *Gdf15*-global-KO animals were randomized into treatment groups based on body weight such that the mean body weights of each group were as close to each other as possible. Oral gavaging was performed at 9:00 AM, GMT, and mice were returned to ad libitum HFD after treatment administration. 1 h after treatment administration, blood was collected in heparinized capillary tubes via the tail vein (approximately 50 μL) after which animals were returned to their cages for a 1-week washout period. Following blood collection, capillaries were immediately placed on ice and spun for 4 min at 10,000 RPM to isolate plasma before being frozen and stored at − 80 °C until assayed. After the wash-out period, animals received the same treatment (although metformin groups received a 300 mg/kg dose as opposed to 600 mg/kg) again via oral gavage. Oral gavaging was performed at 9:00 AM, GMT, and mice were returned to ad libitum HFD after treatment administration. 10 min after treatment administration, blood was collected in heparinized capillary tubes via the tail vein (approximately 50 μL) after which animals were returned to their cages for 4 h. Following blood collection, capillaries were immediately placed on ice and spun for 10 min at 2,000 g to isolate plasma before being frozen and stored at − 80 °C until assayed. 4 h after treatment administrations, animals were culled by cervical dislocation. Blood was collected, processed, and analyzed for Mouse GDF15 as in MS1. Mouse Total GLP-1 concentrations were measured using a V-PLEX GLP-1 Total Kit assay (Meso Scale Discovery). Mouse PYY concentrations were measured using a R-PLEX Mouse/Rat PYY Total Kit assay (Meso Scale Discovery). Tissues were fresh frozen on dry ice and kept at − 80 °C until the day of RNA extraction.

#### Mouse study 6 (MS6)

##### 15-week high-fat diet, wild-type, *Gdf15*-gut-knockout, and *Gdf15*-global-knockout mice

Experimental cohorts of male *Gdf15*-gut-KO mice were generated as in MS1. C57BL/6N-Gdf15tm1a(KOMP)Wtsi/H mice (referred to as *Gdf15*-global-KO mice) were obtained from the MRC Harwell Institute, which distributes these mice on behalf of the European Mouse Mutant Archive (https://www.infrafrontier.eu/). Mice were approximately 8 weeks of age when transferred from a standard chow diet to a 60% high-fat diet (HFD; Research Diet Inc. no. 12492i) for 15 weeks. While fed an HFD, the body weight of each mouse was monitored weekly. Animals were single-housed after 13 weeks on high-fat diet. After 15 weeks on high-fat diet, mice were culled by cervical dislocation. Blood was collected, processed, and analyzed for Mouse GDF15 as in MS1. Mouse FGF21 levels were measured using a Mouse/Rat FGF21 Quantikine ELISA Kit (R&D Systems) which had been modified to run as an electrochemiluminescence assay on the Meso Scale Discovery assay platform. Tissues were fresh frozen on dry ice and kept at − 80 °C until the day of RNA extraction.

### Experimental diets

Mice were given ad-libitum access to either standard laboratory chow (RM3(E) Expanded Chow (Special Diets Services)), a 60% high-fat diet (Research Diet Inc. no. 12492i), or a high-fat high-sucrose diet (ResearchDiets no. 20022503). Each diet’s composition is outlined in Tables 1, 2 and 3, respectively. The specific diet used was outlined in each mouse study (MS1-6).

**Table 1:** Composition of chow diet used in mouse studies.

Chow diet (CD)	Value	Source
AFE (kcal/g)	3.63	
Energy (% kcal)		
Fat	11.5	Soybean oil
Protein	27	Amino acid blend
Carbohydrate	61.5	7:1 maltodextrin:sucrose

*AFE* Atwater free energy.

**Table 2:** Composition of high-fat diet used in mouse studies.

High-fat diet (HFD)	Value	Source
AFE (kcal/g)	5.24	
Energy (% kcal)		
Fat	60	10:1 lard:soybean oil
Protein	20	Casein (+ L-cystine)
Carbohydrate	20	2:1 maltodextrin:sucrose

*AFE* Atwater free energy.

**Table 3:** Composition of HFHS diet used in mouse studies.

High-fat high-sucrose diet	Value	Source
AFE (kcal/g)	4.6	
Energy (% kcal)		
Fat	41	6.4:1 lard:soybean oil
Protein	20	Casein (+ L-cystine)
Carbohydrate	39	1:3 maltodextrin:sucrose

*AFE* Atwater free energy.

### In situ hybridization

Tissues were dissected and placed into 10% formalin for 48 h at room temperature, transferred to 70% ethanol and embedded into paraffin. Five-micrometre sections were cut using a Leica microtome, mounted onto Superfrost Plus slides (Thermo Fisher Scientific) and stained for hematoxylin and eosin. Detection of *Cre* mRNA was performed on formalin-fixed paraffin-embedded (FFPE) sections, obtained from 60% high-fat diet-fed GDF15^flox/flox^-Villin-Cre^-^ (wild-type) and GDF15^flox/flox^-Villin-Cre^+^ (*Gdf15*-gut-KO) (MS1; Section 2.1.2), using Advanced Cell Diagnostics (ACD) RNAscope^®^ 2.5 LS Reagent Kit-RED (no. 322150) and RNAscope^®^ 2.5 LS Probe CRE-C2 (ACD, no. 312288-C2). Slides were processed as previously described^3^. Positive and negative controls were run in parallel each time. To prevent the *Cre* probe from binding to genomic DNA (where necessary), slides were treated with DNase I (QIAGEN, no. 79154) for 10 min at room temperature prior to probe incubation.

Detection of Mouse *Gdf15* was performed on FFPE sections using Advanced Cell Diagnostics RNAscope® 2.5 LS Reagent Kit-RED (ACD, no. 322150) and RNAscope^®^ LS 2.5 Probe Mm-Gdf15-O1 (ACD, no. 442948). Fast red detection of mouse *Gdf15* was performed on the Bond RX using the Bond Polymer Refine Red Detection Kit (Leica Biosystems, no. DS9390) according to the ACD protocol. Slides were then counterstained with haematoxylin, removed from the Bond RX and were heated at 60 °C for 1 h, dipped in Xylene and mounted using EcoMount Mounting Medium (Biocare Medical, CA, USA. no. EM897L).

All slides were imaged using the Zeiss AxioScan.Z1 Slide Scanner at × 20 (standard) or × 40 magnification (RNAscope). For the RNAscope slides, three Z-stacks spanning a total of 2 μm were acquired and merged into a single extended depth of focus (EDF) image with maximum projection processing, and then sharpened using Unsharp Masking (ZEN Blue, Zeiss).

### RNA isolation, cDNA synthesis, and qPCR

RNA extraction was carried out with approximately 100 mg of tissue in 1 ml Qiazol Lysis Reagent (Qiagen 79306l) using Lysing Matrix D homogenisation tube, Fastprep 24 Homogenizer (MP Biomedicals), and Qiagen RNeasy Mini Kit (no. 74106) following manufacturers’ protocols. RNA concentration and quality was determined by Nanodrop spectrophotometry. 400–500 ng of total RNA was then converted to cDNA using M-MLV Reverse Transcriptase with random primers (Promega). Quantitative RT–PCR was carried out with either TaqMan Universal PCR Master Mix or SYBR Green PCR master mix on the QuantStudio 7 Flex Real time PCR system (Applied Biosystems). All reactions were carried out in triplicate and Ct values were obtained. Relative differences in gene expression were normalized to the expression levels of the housekeeping gene Actin Beta (*Actb*) using the standard curve method. TaqMan probes used for these studies: mouse *Gdf15* (Mm00442228_m1, ThermoFisher Scientific) and ActB (Mm02619580_g1, ThermoFisher Scientific).

### Statistical analyses

GraphPad Prism (Version 9, GraphPad Software Inc., San Diego, CA) software was used for data visualization and statistical analyses. Unpaired Student’s t-test, two-way ANOVA, and three-way ANOVA with multiple comparison adjustment by Tukey’s or Sidak’s test were used for comparison of differences between experimental groups. Analysis of Covariance (ANCOVA) was performed using JASP (Version 0.16.3, JASP Team, Amsterdam, Netherlands) to determine metabolic rate with body weight as a covariate, genotype as a fixed factor, and energy expenditure as the dependent variable. The specific statistical method employed for individual data sets is listed in the figure legends.

### Supplementary Information

Supplementary Information.

## Data Availability

Data will be made available from the corresponding author upon request.
