# Supplementary material for: The gastrointestinal tract is a major source of the acute metformin-stimulated rise in GDF15
Source: Sci Rep. 2024 Jan 22;14:1899. doi: 10.1038/s41598-024-51866-2 (PMC10803367; doi:10.1038/s41598-024-51866-2)
Supplement: Supplementary file 1 — Supplementary Information. [file 41598_2024_51866_MOESM1_ESM.docx]

**Appendix A. Supplementary Figures**


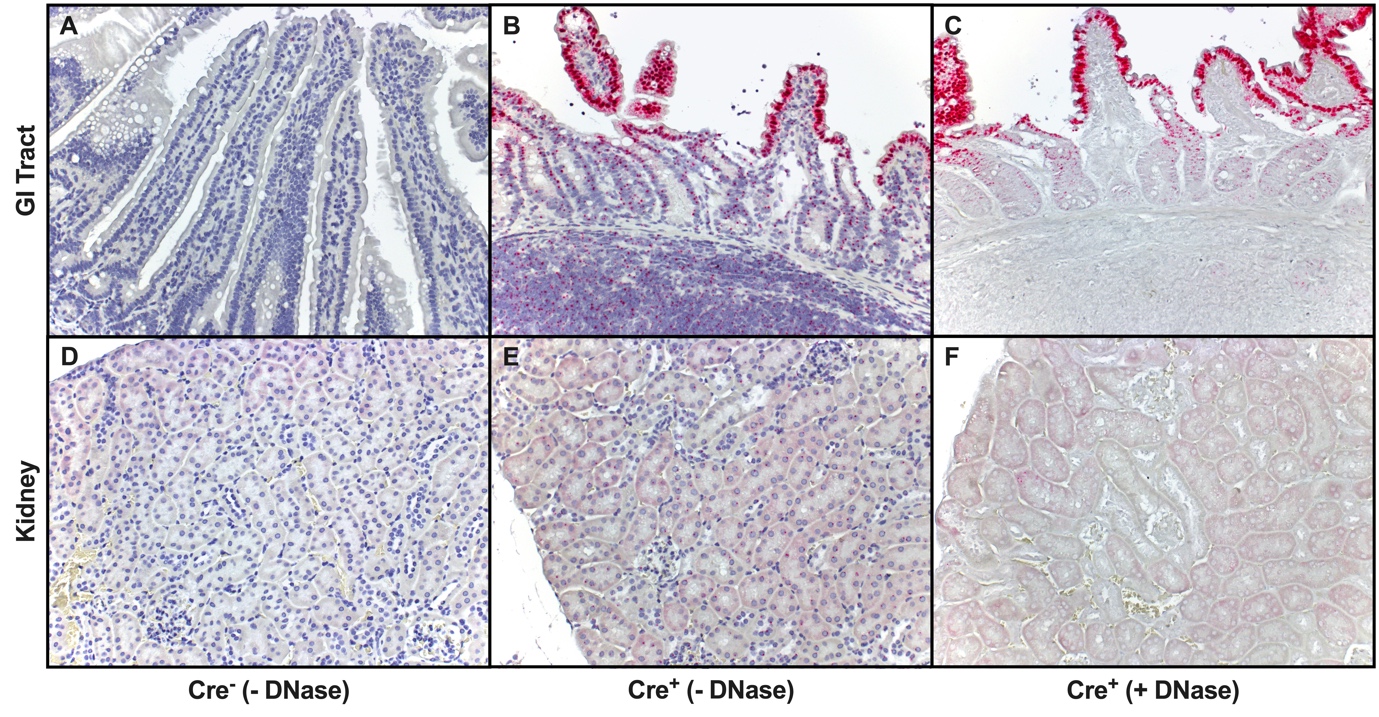


Supplementary Figure A.1. *Cre* RNAscope of the intestine and kidney of C57Bl/6N-Gdf15^flox/flox^-Villin-Cre^-^ (Cre^-^) and C57Bl/6N-Gdf15^flox/flox^-Villin-Cre^+^ (*Gdf15*-gut-KO) mice**.** Detection of *Cre* mRNA using RNAscope in **(A)** small intestinal and **(D)** kidney tissue of 4-week HFD-fed wild-type (Cre^-^) mice, **(B)** small intestinal and **(E)** kidney tissue of Gdf15^flox/flox^-Villin-Cre^+^ (*Gdf15*-gut-KO) mice, and DNase-treated **(C)** small intestinal and **(F)** kidney tissue of 4-week HFD-fed Gdf15^flox/flox^-Villin-Cre^+^ (*Gdf15*-gut-KO) mice.


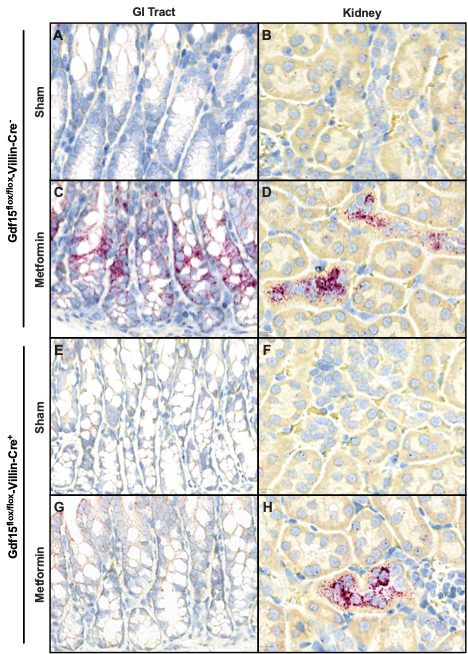


Supplementary Figure A.2. *Gdf15* RNAscope of the intestine and kidney of C57Bl/6N-Gdf15^flox/flox^-Villin-Cre^-^ (Cre^-^) and C57Bl/6N-Gdf15^flox/flox^-Villin-Cre^+^ (*Gdf15*-gut-KO) mice**.** Detection of *Gdf15* mRNA using RNAscope in **(A)** colonic and **(B)** renal tissue of sham-treated, 4-week HFD-fed wild-type mice, **(C)** colon and **(D)** renal tissue of metformin-treated wild-type mice, **(E)** colonic and **(F)** renal tissue of sham-treated, 4-week HFD-fed Gdf15^flox/flox^-Villin-Cre^+^ (Gdf15-*gut*-KO) mice, and **(G)** colonic and **(H)** renal tissue of metformin-treated *Gdf15*-gut-KO mice.


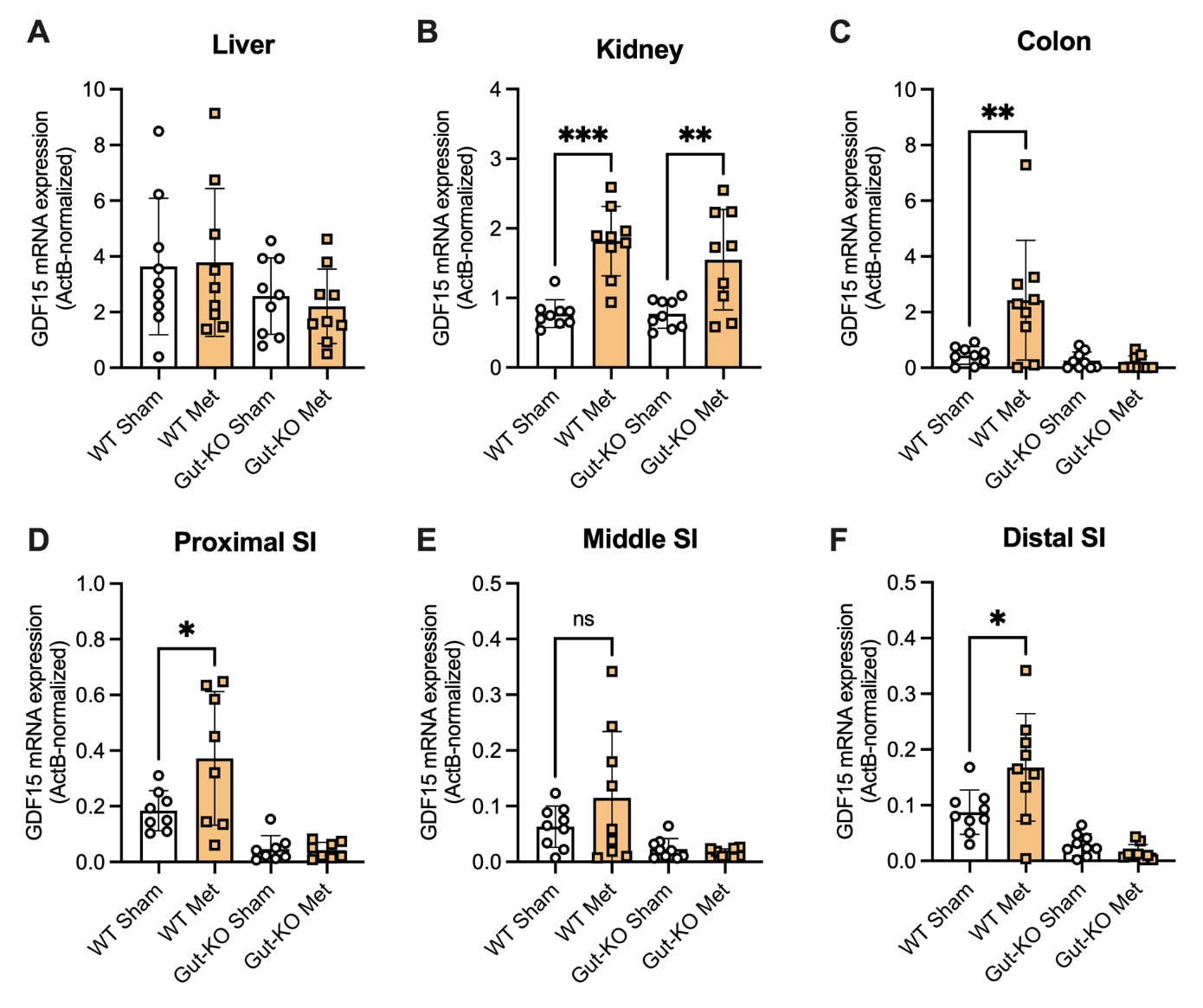


Supplementary Figure A.3. Metformin-induced rises in circulating GDF15 are diminished in chow-fed *Gdf15*-gut-KO mice**.** (A-F) *Gdf15* mRNA expression (normalized to expression levels of actin B) in the **(A)** liver, **(B)** kidney, **(C)** colon, **(D)** proximal small intestine, **(E)** middle small intestine, and **(F)** distal small intestine from chow-fed wild-type and *Gdf15*-gut-KO mice 6 hours after receiving a single oral dose of water (Sham) or 600 mg/kg metformin (Met). n = 9/group, mean ± SEM, and *p < 0.05, **p < 0.01, and ***p < 0.001 as determined by two-way ANOVA.


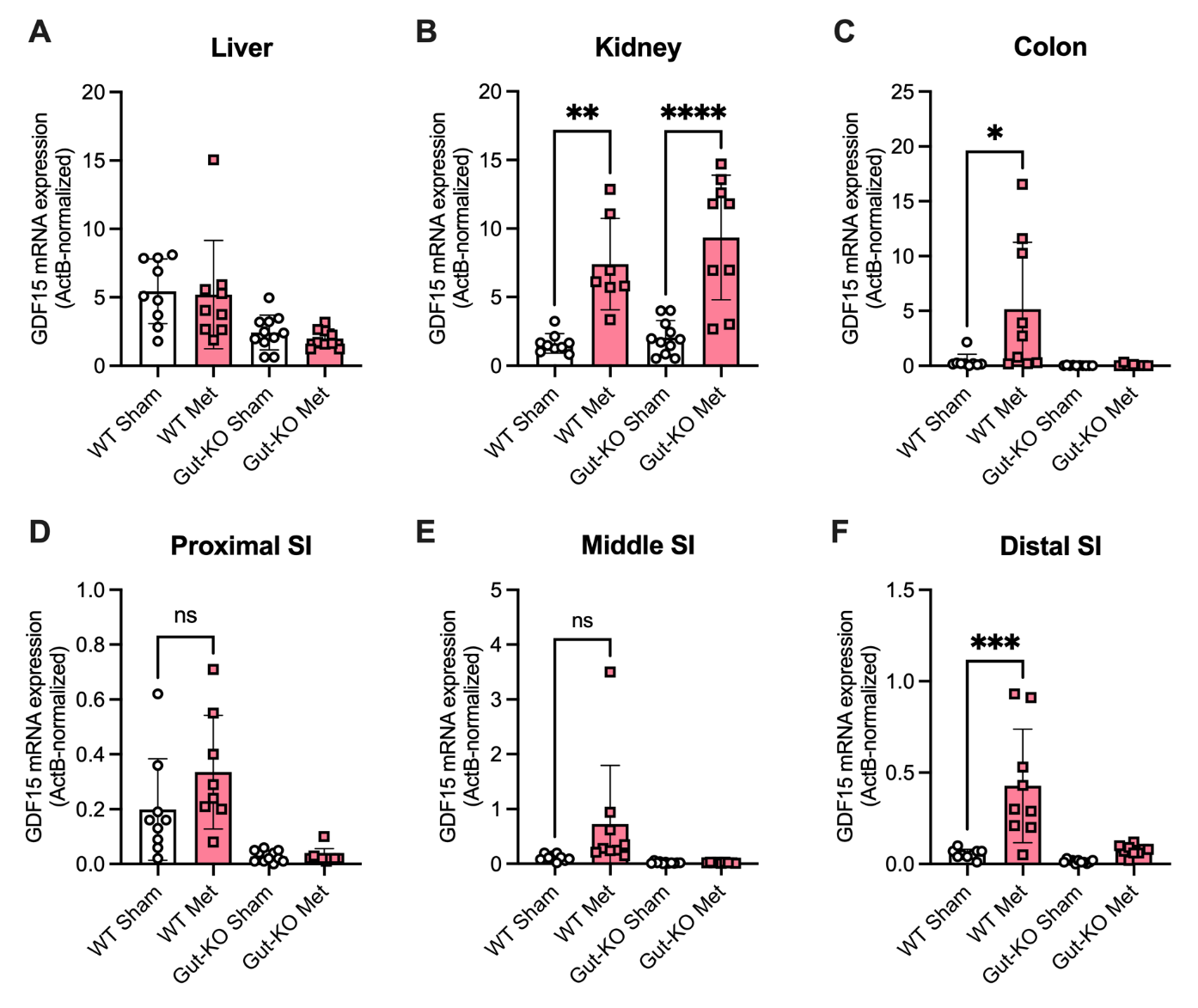


Supplementary Figure A.4. HFHS diet feeding potentiates the metformin-stimulated rise in GDF15 in wild-type and *Gdf15*-gut-KO mice**.** (A-F) *Gdf15* mRNA expression (normalized to expression levels of actin B) in the **(A)** liver, **(B)** kidney, **(C)** colon, **(D)** proximal small intestine, **(E)** middle small intestine, and **(F)** distal small intestine from HFHS diet-fed wild-type and *Gdf15*-gut-KO mice 6 hours after receiving a single oral dose of water (Sham) or 600 mg/kg metformin (Met). n = 9/group, mean ± SEM, and *p < 0.05, **p < 0.01, ***p < 0.001, and ****p<0.0001 as determined by two-way ANOVA.


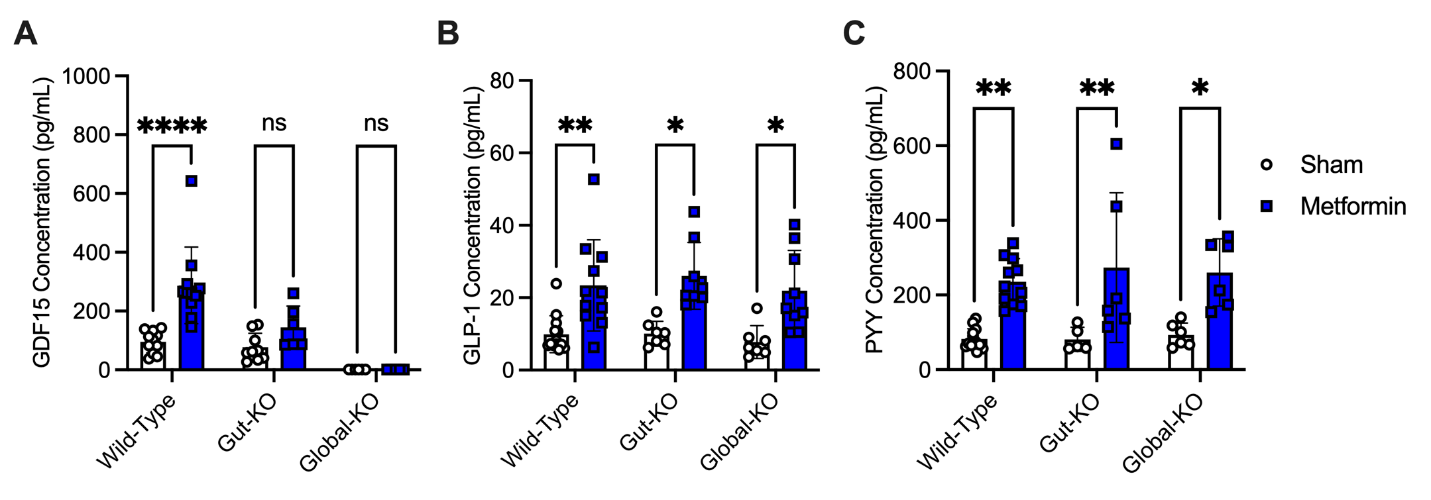


**Supplementary Figure A.5. GDF15, GLP-1, and PYY serology in HFD-fed wild-type, *Gdf15*-gut-KO, and *Gdf15*-global-KO mice given metformin. (A)** Serum GDF15 levels (mean ± SEM) 4 hours after receiving a single oral dose of water (sham) or 300 mg/kg metformin in 12-week-old wild-type, *Gdf15*-gut-KO, and *Gdf15*-global-KO mice fed a 60% high-fat diet for 4 weeks, n = 11/group for wild-type and *Gdf15*-gut-KO and n = 6/group for *Gdf15*-global-KO mice, and ****p<0.0001 as determined by two-way ANOVA. **(B)** Serum GLP-1 levels (mean ± SEM) 10 minutes after receiving a single oral dose of water (sham) or 300 mg/kg metformin in 12-week-old wild-type, *Gdf15*-gut-KO, and *Gdf15*-global-KO mice fed a 60% high-fat diet for 5 weeks, n = 11/group for wild-type and *Gdf15*-gut-KO and n = 6/group for *Gdf15*-global-KO mice, and **p<0.01 as determined by two-way ANOVA. **(C)** Serum PYY levels (mean ± SEM) 1 hour after receiving a single oral dose of water (sham) or 600 mg/kg metformin in 12-week-old wild-type, *Gdf15*-gut-KO, and *Gdf15*-global-KO mice fed a 60% high-fat diet for 5 weeks, n = 13/group for wild-type and n = 6/group for *Gdf15*-gut-KO and *Gdf15*-global-KO mice, and ****p<0.0001 as determined by two-way ANOVA.
